# Can we identify women who initiate and then prematurely cease breastfeeding? An Australian multicentre cohort study

**DOI:** 10.1186/s13006-015-0040-y

**Published:** 2015-05-04

**Authors:** Julie Quinlivan, Sonia Kua, Robert Gibson, Andrew McPhee, Maria M Makrides

**Affiliations:** Institute for Health Research, University of Notre Dame Australia, Fremantle, 6160 WA Australia; King Edward Memorial Hospital, Subiaco, 6008 WA Australia; Women’s and Children’s Health Research Institute, Adelaide, 5000 SA Australia

**Keywords:** Breastfeeding, Pregnancy, Longitudinal study, Education, Preterm birth, Low birth weight, Smoking

## Abstract

**Background:**

Health authorities recommend 6 months of fully breastfeeding and continuation of breastfeeding for at least a year. Many women initiate breastfeeding in hospital but discontinue before the six-month period, and therefore do not optimise the public health benefits. The aim of this study was to determine whether these women could be identified at hospital discharge, to enable targeted interventions.

**Methods:**

A secondary analysis of women who intended to breastfeed and were enrolled in a large randomised trial was undertaken. Women were enrolled in the antenatal period and antenatal, delivery and six month postnatal questionnaires were completed. Univariate and multivariate analyses were undertaken to determine the variables associated with early cessation of breastfeeding within six months, compared to women who continued to breastfeed.

**Results:**

Of 2148 women who initiated breastfeeding in hospital, 877 continued to breastfed either partially (N = 262) or fully (N = 615) until six months postpartum and 1271 ceased breastfeeding early. Median breastfeeding duration in women who ceased early was 3^+6^ weeks (IQR 1^+1^ to 11^+2^ weeks). In multivariate analysis, factors that were significantly associated with early cessation of breastfeeding were maternal factors of lower education (less than 12 years of schooling, no completion of further education), smoking (pre-pregnancy or during pregnancy), and newborn factors of preterm birth and low birthweight (all p < 0.01). These variables correctly identify 83% of women.

**Conclusion:**

We can identify women who initiate and then prematurely discontinue breastfeeding prior to hospital discharge. Evaluation of additional interventions to support longer duration of breastfeeding in women at risk of ceasing prematurely is needed.

## Background

The World Health Organization and National Health and Medical Research Council Australia (NHMRC) recommend fully breastfeeding infants until 6 months, with continuation of breastfeeding to 12 months of age [1,2]. Whilst the majority of Australian babies are breastfed when discharged from hospital (92.3%), by six months rates of breastfeeding have dropped to 50.2% [3]. A similar story of high initiation, followed by high early cessation is reported globally in the developed world [4]. In China, 98% of women initiate breastfeeding, but only 50% still breastfeed at six months, and in only 20% is breastfeeding exclusive [5].

Human breast milk is species-specific and is associated with benefits to both mother and child [1]. The newborn benefits include a protective effect against infections (bacterial meningitis, gastrointestinal, respiratory tract, urinary tract, otitis media) and decreased morbidity from atopic disease (dermatitis, asthma)[1,4-6]. Long-term benefits to the breastfed child include a reduction in both type 1 and type 2 diabetes mellitus, obesity and reduced rates of lymphoma and leukaemia in adult life [1,6].

Breastfeeding also confers benefits to the mother. Short-term benefits include reduced postpartum bleeding and increased postpartum weight loss [1,7]. Prolonged lactational amenorrhoea leads to greater intervals between children. Long-term benefits to the mother include a reduced risk of ovarian and post-menopausal breast cancer, and a possible reduction in risk of postmenopausal osteoporosis and hip fracture [1,4].

The positive public health impact of breastfeeding upon subsequent obesity risk in the mother and her offspring is of manifest importance as global rates soar [8].

Early discontinuation of breastfeeding neutralises many of the potential benefits, especially in regard to maternal weight loss in the first postnatal years. If we could clearly identify women who plan to breastfeed but then prematurely cease before six months, we could develop targeted interventions for this cohort. These women display a desire to breastfeed, and therefore interventions are likely to be welcome.

The aim of the present study was to identify those factors associated with cessation of breastfeeding before six months postpartum and determine if this helped identify women who prematurely cease breastfeeding after successful initiation in hospital.

## Methods

### Study design

We employed data from a subset of women enrolled in a RCT named *DHA to Optimize Mother and Infant Outcome* (DOMInO). The DOMInO trial involved 2399 pregnant women, recruited from 2005 to 2008, at <20 week gestation with singleton pregnancies. DOMInO was a double-blind, multicentre RCT conducted in five Australian perinatal centres: two in South Australia and one each in New South Wales, Victoria and Queensland [9].

Study participants analysed in the present manuscript were the subset of women from the DOMInO trial who delivered at baby friendly hospitals and were certified in their medical record as competently breastfeeding their infant at discharge by their attending midwife. These subjects were followed until six months postpartum as part of the DOMInO study protocol. They were telephoned at six weeks and six months postpartum by a study nurse and interviewed. Information collected at interview included infant feeding status, in particular, whether they were fully or partially breastfeeding their infant and whether they had introduced any solids.

### Study hypothesis and sample size

Our hypothesis was that lower level of maternal education would be a leading predictor of early discontinuation of breastfeeding in line with the largest previously published prospective study [10]. We predicted that 70% of women who continued to breastfeed to 6 months would have achieved post secondary education, against a background rate of 60% in women using formula. To achieve power of 90% and alpha error of 0.05, a sample size of 496 was required in each group.

### Classification of infant feeding

A research nurse interviewed each study participant at six weeks and six months postpartum. Based on information supplied by the mother at these assessments, feeding was coded as ‘breastfeeding’ if the mother reported that she continued to breastfeed the baby or breastfed in combination with infant formula at six months. If the mother reported that she exclusively infant formula fed, then feeding was coded as ‘formula’. Fully breastfeeding was defined as breastfeeding with no infant formula or solids, although administration of water and juices was allowed in the preceding 24 hours.

### Antenatal assessments

Baseline demographic and obstetric characteristics of the mother and assessments of social support, depressive symptomatology, smoking and alcohol use were established using validated tools that have been previously utilised in maternal and child health studies [11-19].

### Postnatal assessments

Pregnancy data in respect to infant gender, gestational age at birth, preterm birth, very preterm birth and birth weight were abstracted from the hospital medical record. Symptoms of postnatal depression were assessed using the Edinburgh Postnatal Depression Scale (EPDS), which was completed by mothers at six weeks and six months post-partum. A score of more than 12 was coded as a positive case for depression [17-19].

A trial coordinator monitored data collection, which was facilitated by a web-based management information system.

### Study analysis

Discrete variables were presented as number and percentage and were analysed using Chi Square tests to generate p-values and Odd Ratio with 95% confidence intervals. Continuous variables with normal distribution were presented as mean and standard deviation and compared using T-tests.

Continuous variables with skewed distribution were presented as median and interquartile range and compared using Mann Whitney U tests.

Factors significantly associated with feeding type at a p-value <0.1 were included in a multivariate regression model. Statistical significance was accepted at the 0.05 levels. We then retrospectively applied a model of significant independent associations to determine the extent to which these variables identified women who prematurely ceased breastfeeding.

### Ethics approvals

Ethics Committees conformed to NHMRC and Declaration of Helsinki 1995 criteria and prospective trial registration was undertaken. The Ethics Committees and approval numbers were: Women’s and Children’s Hospital, South Australia, Australia - 1657/12/2007; Flinders Medical Centre, South Australia, Australia - 199/045; Sunshine Hospital, Victoria, Australia - REC 1657/12/2010; Campbelltown Hospital, New South Wales, Australia - HE05/142; and Royal Brisbane and Women's Hospital, Queensland, Australia – 2007000424.

Written informed consent was obtained from each participant. Data in this manuscript were extracted from the DOMInO trial data set and were subjected to secondary analysis. The DOMINO trial was registered on the Australian Clinical Trials Research Network (ACTRN12605000569606) [9].

## Results

Of the 2399 women enrolled in the original study, 2148 (89.5%) women initiated breastfeeding in hospital. This subgroup of 2148 women were analysed in the present report. Postnatal assessments were made at a mean postpartum age of 25.9 weeks (sd 1.4 weeks).

A total of 877 women continued to breastfeed either partially (N = 262) or fully (N = 615) until six months postpartum. The results of the women who partially or fully breastfed for six months (breastfed group) were subsequently compared to those women who discontinued breastfeeding early and were exclusively infant formula feeding (formula) at six months (N = 1271). Median duration of breastfeeding in women who ceased breastfeeding prior to six months was 3^+6^ weeks (IQR 1^+1^ to 11^+2^ weeks).

Table 1 summarises the antenatal demographic characteristics of the women who continued to breastfeed for six months (breastfeeding group) compared to those who discontinued early (early cessation group). Antenatal variables significantly associated with early cessation of breastfeeding on univariate analysis were younger maternal age (p < 0.0001), less than 12 years of schooling (p < 0.0001), no completion of further education (p < 0.0001), a past history of maternal depression (p = 0.004), and maternal self-identification as a smoker pre-pregnancy (p < 0.0001) or during pregnancy (p < 0.0001).

**Table 1:** **Antenatal demographic characteristics of the women who continued to breastfeed for six months (breastfeeding group) compared to those who discontinued before six months postnatal (early cessation group)**

**Variable**	**Early cessation group N=1271**	**Breastfeeding group N=877**	**P-value OR (95% CI)**
Age Mean (sd)	28.0 (5.6)	30.3 (5.3)	<0.0001
Country of birth N (%)			0.87
Australia	1169 (91.9%)	777 (88.6%)	
Asia	68 (5.4%)	77 (8.8%)	
Other	34 (2.7%)	23 (2.6%)	
Aboriginal or torres strait islander N (%)			0.45
Yes	26 (2.0%)	8 (0.9%)	
No	1245 (98.0%)	869 (99.1%)	
Gravidity Median (IQR)	1 (0-2)	1 (0-2)	0.73
Parity Median (IQR)	1 (0-1)	1 (0-2)	0.11
Completed secondary education N (%)			
Yes	691 (54.4%)	683 (77.9%)	<0.0001
No	580 (45.6%)	194 (22.1%)	0.34 (0.28, 0.41)
Completed further education N (%)			
Yes	628 (49.4%)	665 (75.8%)	<0.0001
No	643 (50.6%)	211 (24.1%)	0.40 (0.33, 0.48)
Missing data		1 (0.1%)	
History of depression N (%)			
Yes	335 (26.4%)	184 (21%)	0.004
No	936 (73.6%)	693 (79%)	1.35 (1.09, 1.66)
Maternal social support index score			
Mean (sd)	27.6 (4.9)	28.0 (4.8)	0.06
Pre-pregnancy smoker N (%)			
Yes	509 (40.1%)	161 (18.4%)	<0.0001
No	762 (59.9%)	715 (81.5%)	2.97 (2.41, 3.66)
Missing data	0	1 (0.1%)	
Smoking in pregnancy N (%)			
Yes	280 (22.0%)	65 (7.4%)	<0.0001
No	991 (78.0%)	812 (92.6%)	3.53 (2.63, 4.74)
Pre-pregnancy alcohol N (%)			
Yes	735 (57.8%)	523 (59.6%)	0.43
No	536 (42.2%)	354 (40.4%)	0.93 (0.78, 1.11)
Alcohol in pregnancy N (%)			
Yes	116 (9.1%)	73 (8.3%)	0.51
No	1155 (90.9%)	804 (91.7%)	1.11 (0.81, 1.52)

Table 2 summarises infant and postnatal factors in the breastfeeding group compared to the early cessation group. Factors significantly associated with early cessation of breastfeeding on univariate analysis were preterm birth (p = 0.004), very preterm birth (p = 0.026), and low birth weight (less than 10^th^ centile adjusted for gestational age) (p < 0.0001).

**Table 2:** **Newborn and postnatal variable outcomes in women who continued to breastfeed for six months (breastfeeding group) compared to those who discontinued before six months postnatal (early cessation group)**

**Variable**	**Early cessation group N=1271**	**Breastfeeding group N=877**	**P-value OR (95% CI)**
Baby gender N(%)			
Male	647 (50.9%)	433 (49.4%)	0.49
Female	624 (49.1%)	444 (50.6%)	1.06 (0.89, 1.27)
Gestational age at birth (days)			
Mean (sd)	275 (13)	277 (10)	0.17
Preterm birth (<37 wks) N (%)			
Yes	90 (7.1%)	36 (4.1%)	0.004
No	1181 (92.9%)	841 (95.9%)	1.78 (1.18, 2.70)
Late preterm birth (34^+1^ to 36^+6^ wks) N (%)			
Yes	67 (5.4%)	30 (3.4%)	0.05
No	1181 (94.6%)	841 (96.6%)	1.59 (1.02, 2.47)
Very preterm birth (<34 wks) N (%)			
Yes	23 (1.8%)	6 (0.7%)	0.026
No	1248 (98.2%)	871 (99.3%)	2.68 (1.03, 7.35)
Birthweight (gms)			
Mean (sd)	3399 (571)	3532 (498)	<0.0001
Edinburgh postnatal depression scale case*			
at 6 weeks N (%)			
Yes	125 (9.8%)	67 (7.6%)	0.07
No	1130 (88.9%)	807 (92.0%)	1.33 (0.97, 1.84)
Missing data	16 (1.3%)	3 (0.3%)	
Edinburgh postnatal depression scale case*			
at 6 months N (%)			
Yes	132 (10.4%)	90 (10.3%)	0.90
No	1134 (89.2%)	787 (89.7%)	1.02 (0.76, 1.36)
Missing data	5 (0.4%)	0 (0%)	

*A Edinburgh Postnatal Depression ScaleCase was an individual whose score was more than 12.

Table 3 summarises the multivariate analysis. In multivariate analysis, factors that remained significantly associated with early cessation of breastfeeding were less than 12 years of schooling (p < 0.0001), no completion of further education (p < 0.0001), smoking before pregnancy (p < 0.0001) or during pregnancy (p < 0.0001), preterm birth (P = 0.01) and lower birthweight (p < 0.0004).

**Table 3:** **Multivariate analysis of factors associated with early cessation of breastfeeding**

**Significant associations and predictors**	**P > z**	**Difference (95% CI)**
Incomplete secondary education	<0.0001	+21% (+17, +25)
No additional education	<0.0001	+20% (+16, +24)
Smoking prior to pregnancy	<0.0001	+20% (+16, +24)
Smoking during pregnancy	<0.0001	+13% (+11, +150
Low birth weight	0.0004	-132 g (-206, -58)
Preterm birth	0.01	+3% (+1, +5)
Very preterm birth	0.09	+0.5% (-0.4, +1.4)
Maternal postnatal depression	0.12	+2.1% (-0.6, +4.8)
Maternal age	0.15	-2.0 yrs (-4.9, +0.9)
Maternal antenatal depression	0.25	+4.1% (-2.1, +6.2)
Maternal social support index	0.32	-0.4% (-2.4, +1.6)

In retrospectively applying these variables to the cohort, 83% of women who initiated and then prematurely ceased breastfeeding before six months could be identified at the point of hospital discharge.

## Discussion

This is the first large multicentre prospective Australian study to explore the factors associated with early cessation of breastfeeding before six months. The key findings of the study were that the maternal demographic factors of education and smoking status, and the newborn variables of prematurity and birth weight exerted an independent effect upon breastfeeding duration. Half of the women who ceased breastfeeding early had done so within four weeks of the baby’s birth, indicating that this remains a critical period in order to achieve public health breastfeeding goals.

Maternal education has previously been positively linked to breastfeeding [10,20,21]. A study of 11 286 women using data from the Millennium Study linked breastfeeding with educational levels of mothers [20]. A previous Australian study pooling data from three arms of a randomised trial and a large Swedish study also reported an association with maternal education [10,20].

Smoking has also been previously linked to adverse breastfeeding outcomes. A retrospective study reported smoking status impacted upon breastfeeding duration [10]. A large population study from the United Kingdom reported that maternal smoking in pregnancy was the variable with the highest odds ratios for early cessation of breastfeeding [22]. However the effect was mediated largely through a lower motivation to breastfeed rather than a physiological effect of smoking on milk supply [22]. Other studies have also suggested a lack of causality between maternal smoking and breastfeeding and have argued smoking is a marker for low motivation to breastfeed rather than a mediator of altered breast milk composition and volume [23,24].

Our study also evaluated infant factors. Early discontinuation of breastfeeding was significantly associated with preterm birth and lower birthweight.

Breastfeeding offers benefits for the premature infant such as reduced incidence of necrotising enterocolitis, decreased length of hospital stay, increased scoring on cognitive and developmental tests and increased visual development when compared to those who are formula fed [25-27]. However barriers faced by mothers of preterm infants include concerns regarding milk production, issues with expressing or obtaining appropriate equipment, difficulty in feeding the infant and transitioning to breast feeding, lack of support by neonatal intensive care staff and maternal illness resulting in delay of breast milk expression [25-27].

The low rates of breastfeeding in preterm and lower birthweight babies observed in our study are consistent with rates reported elsewhere [25-27]. It is important to consider other factors that will help improve persistence in breastfeeding duration, such as timing and number of postnatal visits following discharge, improved community supports, review of existing models of care amongst health care providers, family and the community, and ensuring wide availability of services to women in all areas [26,27].

## Conclusion

Our study indicates that we can identify those women most at risk of initiating and then prematurely discontinuing breastfeeding, prior to their hospital discharge. We need to evaluate interventions to support a longer duration of breastfeeding in women at risk of ceasing prematurely.
